# Optimization of a high-throughput whole blood expression profiling methodology and its application to assess the pharmacodynamics of interferon (IFN) beta-1a or polyethylene glycol-conjugated IFN beta-1a in healthy clinical trial subjects

**DOI:** 10.1186/1756-0500-6-8

**Published:** 2013-01-05

**Authors:** Normand E Allaire, Steven E Bushnell, Jadwiga Bienkowska, Graham Brock, John Carulli

**Affiliations:** 1 Biogen Idec Inc., Genetics and Genomics Group, 14 Cambridge Center, Cambridge, MA 02142, USA; 2 Biogen Idec Inc., Translational Medicine Department, 14 Cambridge Center, Cambridge, MA 02142, USA

## Abstract

**Background:**

Clinical trials offer a unique opportunity to study human disease and response to therapy in a highly controlled setting. The application of high-throughput expression profiling to peripheral blood from clinical trial subjects could facilitate the identification of transcripts that function as prognostic or diagnostic markers of disease or treatment. The paramount issue for these methods is the ability to produce robust, reproducible, and timely mRNA expression profiles from peripheral blood. Single-stranded complementary DNA (sscDNA) targets derived from whole blood exhibit improved detection of transcripts and reduced variance as compared to their complementary RNA counterparts and therefore provide a better option for interrogation of peripheral blood on oligonucleotide arrays. High-throughput microarray technologies such as the high-throughput plate array platform offer several advantages compared with slide- or cartridge-based arrays; however, manufacturer’s protocols do not support the use of sscDNA targets.

**Results:**

We have developed a highly reproducible, high-through put, whole blood expression profiling methodology based on sscDNA and used it to analyze human brain reference RNA and universal human reference RNA samples to identify experimental conditions that most highly correlated with a gold standard quantitative polymerase chain reaction reference dataset. We then utilized the optimized method to analyze whole blood samples from healthy clinical trial subjects treated with different versions of interferon (IFN) beta-1a. Analysis of whole blood samples before and after treatment with intramuscular [IM] IFN beta-1a or polyethylene glycol-conjugated IFN (PEG-IFN) beta-1a under optimized experimental conditions demonstrated that PEG-IFN beta-1a induced a more sustained and prolonged pharmacodynamic response than unmodified IM IFN beta-1a. These results provide validation of the utility of this new methodology and suggest the potential therapeutic benefit of a sustained pharmacodynamic response to PEG-IFN beta-1a.

**Conclusions:**

This novel microarray methodology is ideally suited for utilization in large clinical studies to identify expressed transcripts for the elucidation of disease mechanisms of action and as prognostic, diagnostic, or toxicity markers.

## Background

The study of the blood transcriptome in the context of clinical pharmacogenomics has generated much interest in recent years [1,2]. The cellular and molecular components of peripheral blood exhibit dynamic responsiveness to physiological, environmental, or pathological stimuli and are in contact with nearly every tissue in the body, allowing for assessment of systemic responses to disease or treatment. As such, peripheral blood is a source of clinically accessible diagnostic, prognostic and pharmacodynamic (PD) markers [3,4]. This idea is supported by a growing body of research that describes the identification of expressed transcripts from human and animal peripheral blood that can function as indicators of disease, as prognostic markers of clinical outcome, of risk of toxicity, and as evidence of a therapy’s pharmacodynamic effects [5-8].

The successful use of gene expression microarrays in basic research studies has spawned great interest in the application of this technology to large clinical pharmacogenomics and population-based studies [9-11]. However, microarray cost, the complexity of sample processing and tracking, and practical limitations in sample throughput have restricted its utilization in clinical investigations [12,13]. Microarray manufacturers have responded to these needs with the recent development of higher-throughput solutions such as the high-throughput (HT) plate array or “array of arrays” [14]. This platform was made possible through reduction and optimization of probe content and advances in photonics, collectively enabling the miniaturization and assembly of 96 arrays into the spatial arrangement of a conventional microtiter plate. Our laboratory’s internal validation studies have confirmed that data from the HT plate array platform is highly concordant to that of industry standard cartridge arrays [15].

RNA is often amplified using T7 RNA polymerase-driven *in vitro* transcription (IVT) [16] to produce complementary RNA (cRNA) targets for hybridization to microarrays. However, the high concentration of hemoglobin transcripts in peripheral blood can induce a globin interference effect, effectively reducing a microarray’s detection sensitivity and increasing its signal variability [17]. Although effective methods have been developed to reduce globin interference [18-20], current methods of mitigation also induce variance in microarray results [21].

The challenges associated with utilizing cRNA targets from peripheral blood as probes for microarray investigations have led to the development of alternative methods of amplification and the use of single-stranded complementary DNA (sscDNA) targets from peripheral blood for microarray hybridization [22,23], effectively improving the sensitivity of microarray hybridizations for detecting peripheral blood transcripts. Results from our laboratory’s internal benchmarking experiments analyzing peripheral blood samples have verified that sscDNA targets improve microarray sensitivity and decrease signal variance as compared with cRNA targets analyzed using globin blocking, degradation, and depletion methods (data not shown).

In the current study, we have systematically optimized sscDNA/HT plate array target mass, hybridization parameters and washing parameters using 2 highly characterized test RNAs with the goal of developing a HT methodology for whole blood transcriptional profiling. Comparative analysis of optimization data against peer-reviewed expression array [15] and quantitative polymerase chain reaction (qPCR) [24] datasets were used to select conditions that improved assay reproducibility and sensitivity. The utility of this new array method (BIIB_HT) was also confirmed through analyses of whole blood samples from a clinical trial comparing pharmacodynamic changes following dosing with either interferon (IFN) beta-1a or polyethylene glycol-conjugated IFN (PEG-IFN) beta-1a.

## Results

### Relative variable effect

**Figure 1:**
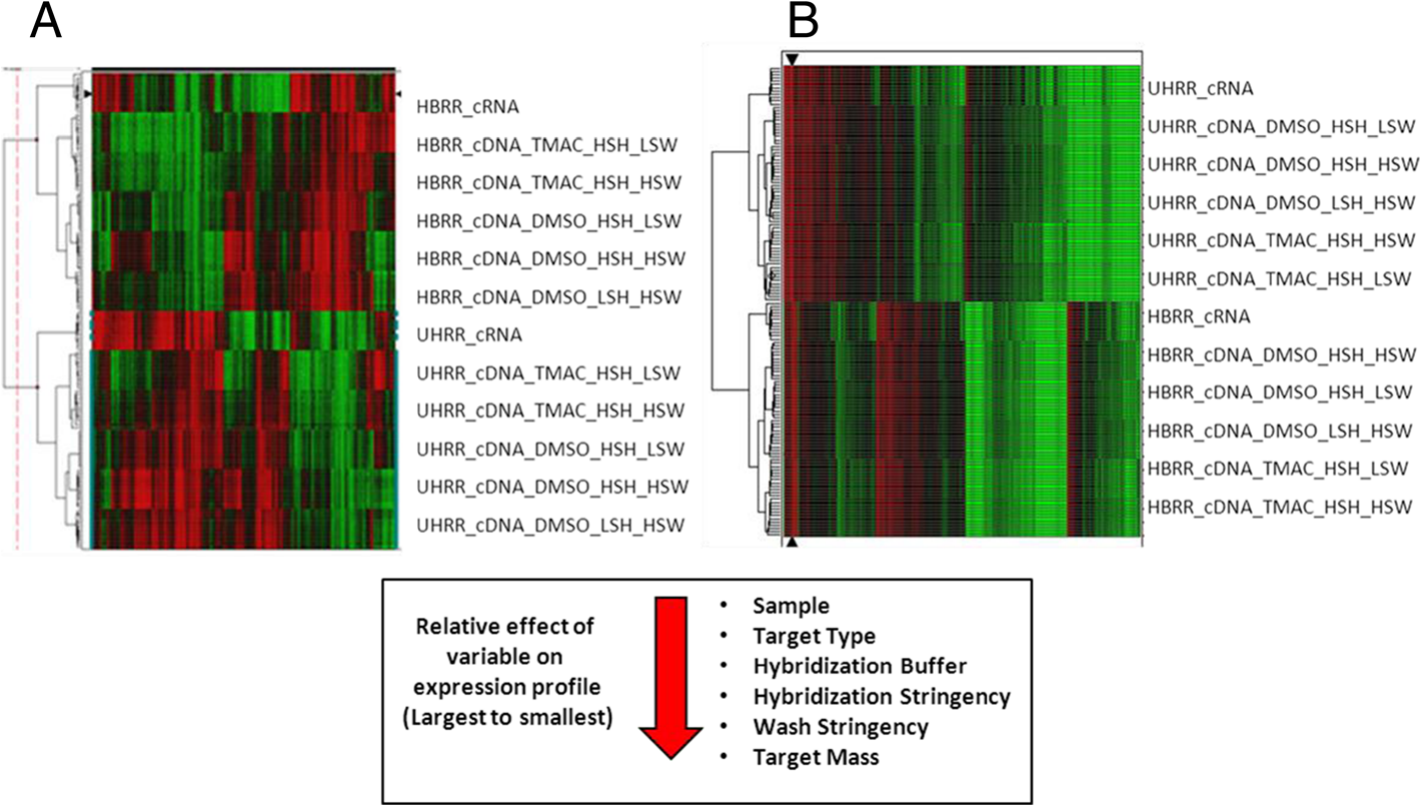
**Comparative analysis of the relative effects of experimental variables on gene expression profiles. A) **An initial filter to remove all qualifiers that changed less than 1.5 fold from the median value in 20% or less of the samples was applied to the dataset. The remaining 13,898 qualifiers were then subjected to hierarchical clustering by correlation with complete linkage. The resultant clustering reflects the experimental conditions that were used in this study. **B) **In initial stringent filter was applied to remove all qualifiers that changed less than 2 fold between human brain reference RNA and universal human reference RNA and a LODS score of >0. The remaining 7128 qualifiers were then subjected to hierarchical clustering using a Euclidean distance measure with single linkage. The experimental conditions are reflected in the clustering dendrogram.

To identify optimal hybridization conditions that both maximize detection of rare transcripts and minimize hybridization variance, labeled sscDNAs generated in bulk from human brain reference RNA (HBRR) and universal human reference RNA (UHRR) were hybridized to HT-HGU133A plate arrays using different masses under varying hybridization and washing conditions. Independent variables for optimization included target type (cDNA and cRNA), mass/array titration (1.0-2.5 μg/array), hybridization cocktail (containing dimethylsulfoxide [DMSO] or tetramethylammonium chloride [TMAC]), hybridization stringency (low-stringency hybridization [LSH, 41-43°C] or high-stringency hybridization [HSH, 48-50°C]), and washing stringency (low-stringency washing [LSW, 41-43°C] or high-stringency washing [HSW, 48-50°C]). Hierarchical analyses were utilized to evaluate the relative impact and rank of the variables tested and to validate the quality of the dataset. An initial filter removed all qualifiers that changed less than 1.5-fold from the median in less than 20% of the data. The remaining 13,898 qualifiers were then clustered by correlation with complete linkage (Figure 1A). We also analyzed the dataset using an alternative filtering and hierarchical clustering approach. We repeated calculations and analyses using R and Bioconductor computational tools as described by Gentleman [25], and to discover qualifiers that were differentially expressed between Human Brain Reference RNA (HBRR) and Universal Human Reference RNA (UHRR) for each condition tested, we applied the linear modeling approach (MANOVA) to fit gene expression levels (log2 transformed) according to the defined groups of samples and Bayesian posterior error analysis as implemented by Smyth [26]. Qualifiers that exhibited a log-odds score (LODS) greater than zero and fold change greater than 2.0 were considered significantly different. This filtering method reduced the number of qualifiers from 13898 to 7128. The remaining 7128 qualifiers were then clustered by single linkage using a Euclidean distance measure or correlation (Figure 1B).

The results from both clustering approaches revealed the significant effect of the experimental conditions on gene expression. Using the derived dendrogram, the relative effects of each condition were ordered from largest to smallest accordingly: sample type > target type > hybridization buffer > hybridization stringency > wash stringency > target mass. Interestingly, target type was second only to sample type in its relative effect on hybridization.

### Global quality assessment

**Figure 2:**
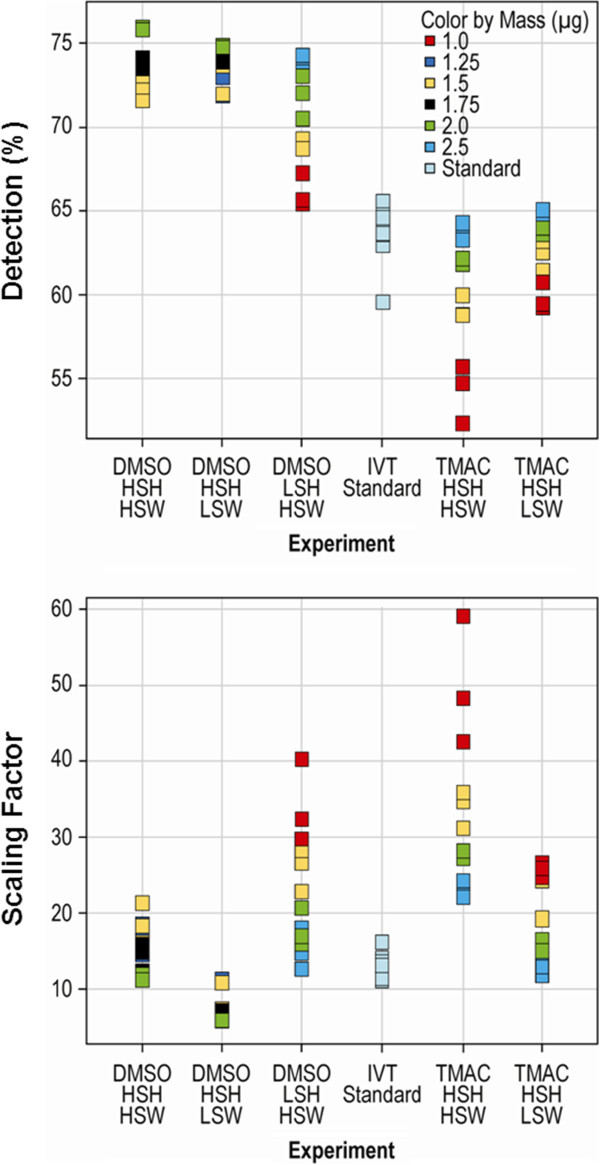
**Global scan quality. **Addition of the cDNA probe in a DMSO buffer increases detection of expressed transcripts.

Initial scan quality was assessed using 2 metrics: the percentage of qualifiers above the background (percent present), and the scaling factor (SF) that was used to adjust the median intensity of the array to a predefined target value. Higher percentages present and lower scaling factors were well correlated with overall scan quality (Figure 2). Results showed that DMSO/sscDNA hybridizations generally resulted in significantly more qualifiers categorized as present than either cRNA or sscDNA targets in native HT TMAC hybridizations (*p* = 4.3 × 10^−23^ and *p* = 1.12 × 10^−23^, respectively), with the sscDNA target mass positively correlated with the percentage present score. Of all DMSO conditions tested, DMSO_HSH_HSW resulted in the highest percentage of qualifiers scored as present, although this percentage was only marginally more than that for DMSO_HSH_LSW (*p* = 0.036). Scaling factor scores for DMSO and TMAC hybridizations were not as well defined. Scaling factors were lowest for sscDNA targets in DMSO_HSH_LSW as compared with all other conditions; however, the lowest scaling factors were produced by sscDNA targets hybridized and washed under the conditions of TMAC_HSH_HSW. As expected, sscDNA target mass was found to be negatively correlated with its scaling factor score.

**Figure 3:**
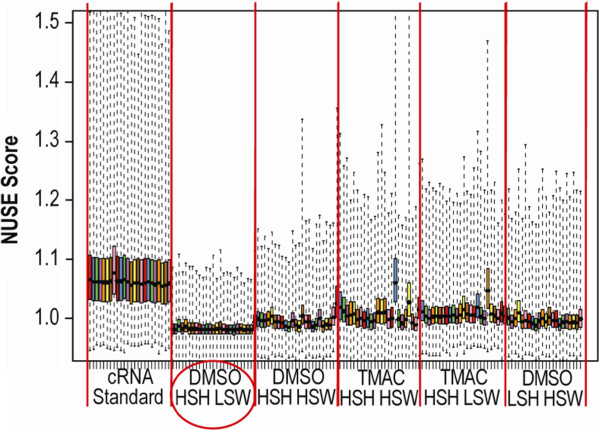
**Standard error plot. **The conditions DMSO HSH_LSW yielded the smallest variance in assay results and was selected as the preferred sample processing methodology.

Normalized, unscaled standard error (NUSE) plots allow for an assessment of variance within an array and for determination of the array’s relationship to a group of arrays [27]. Analysis of cRNA targets hybridized under native HT conditions resulted in the largest relative error among all intra-array and inter-array errors (Figure 3). Conversely, DMSO hybridization resulted in a lower relative error than any native TMAC conditions, with the lowest inter-array and intra-array error produced using sscDNA targets and the hybridization conditions of DMSO_HSH_LSW.

### Data analysis to identify optimal assay conditions

**Figure 4:**
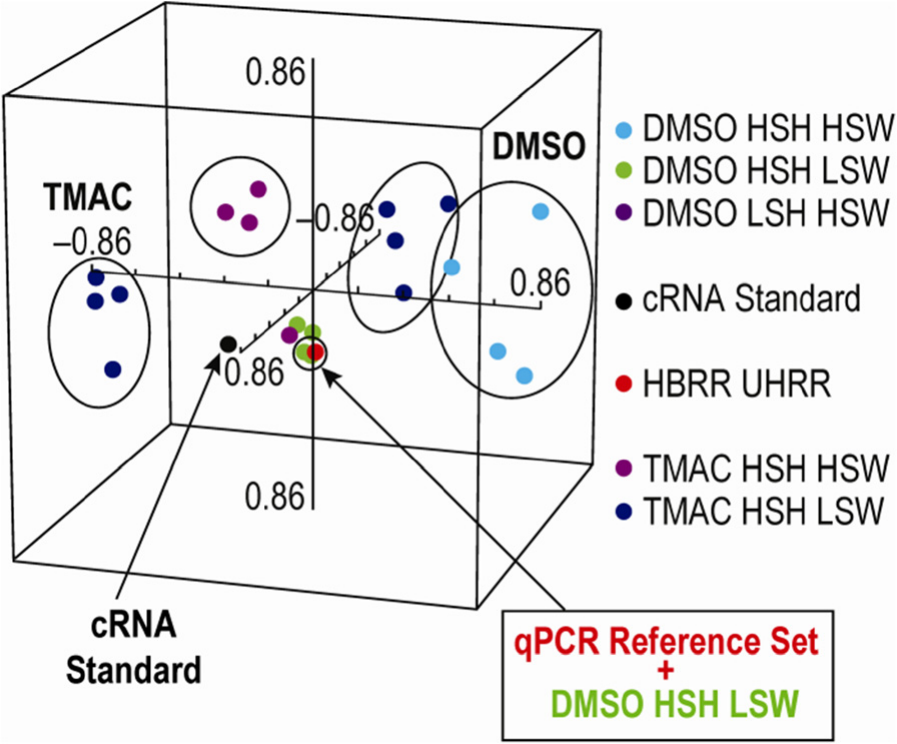
**Principal component analysis. **The BIIB_HT assay expression profile was highly correlated with a qPCR “gold standard”.

The principal component analysis (PCA) method, which reduces the dimensionality of large data sets and allows visualization of the overall data structure, was used to identify experimental HT array hybridization conditions that produced results that were most highly correlated with a gold standard qPCR reference dataset [24]. PCA identified a total of 164 sscDNA qualifier transcripts that changed at least 1.5-fold (*p* = 0.0001) between HBRR and UHRR samples under one of the experimental conditions and that were also present in the qPCR reference dataset. These qualifiers showed a clear separation in clustering between DMSO and TMAC hybridization cocktails (Figure 4). Furthermore, within each cocktail cluster, there was a substructure defined by hybridization and washing stringency that consisted of the all target masses. The qPCR reference clustered most closely with the hybridization conditions of DMSO_HSH_LSW (Figure 4) and these conditions were selected for use with subsequent analyses.

### Detection and differential expression of whole-blood mRNAs encoding IFN beta-1a biological response genes

It has been well established that parenteral administration of IFN beta induces a robust response in systemic gene expression [28]. In order to confirm the utility of this new BIIB_HT method, the optimized assay was utilized for analysis of peripheral blood samples collected as part of a phase 1 clinical dose- and route-finding study [29]. In this study, a single intramuscular (IM) injection of unmodified IFN beta-1a 30 μg was administered and peripheral blood samples were collected predose and at 6 hours and 48 hours postdose. Results showed strong transcript induction at 6 hours (1,398 probe sets, logarithm [base 10] of odds [LOD] score > 0, ±1.5-fold change), with many transcripts returning to pretreatment levels within 48 hours (110 probe sets, LOD score > 0, ±1.5-fold change). A list of the transcripts most commonly upregulated following IFN beta-1a treatment is presented in Table 1. Transcript analysis showed a strong induction of several canonical cell signaling pathways at 6 hours post treatment, including both previously reported and novel candidate pharmacodynamic markers of IFN response. Induced pathways included those involved in IFN signaling, bacterial and virus pattern recognition receptors, IFN regulatory factors, cytoplasmic pattern recognition receptors involved in IFN regulatory factor signaling, and regulation of cytotoxic T lymphocyte-mediated apoptosis (Additional file 1).

**Table 1:** **Whole blood transcripts most frequently upregulated by IFN beta-1a**^**a**^

**Gene Symbol**	**Affymetrix ID**	**Fold Change**	***P *Value**^**b**^	**LOD Score**^**c**^
USP18	219211_at	91.0	1.26E-17	29.6
SIGLEC1	219519_s_at	64.7	2.07E-14	22.3
SPATS2L	222154_s_at	27.2	1.57E-18	31.7
IFI44L	204439_at	26.5	2.54E-14	22.1
HERC5	219863_at	26.2	9.47E-18	29.9
SERPING1	200986_at	23.5	3.28E-12	17.3
RSAD2	213797_at	21.1	6.33E-12	16.7
IFI44	214059_at	20.5	2.70E-13	19.8
OAS3	218400_at	20.3	1.65E-14	22.5
OASL	205660_at	17.7	4.61E-16	26.1
IFIT1	203153_at	15.4	1.59E-13	20.3
RTP4	219684_at	14.6	1.35E-12	18.2
HERC6	219352_at	14.4	1.03E-19	34.4
IFIT3	204747_at	13.8	2.90E-14	22.0
OAS1	205552_s_at	13.3	7.93E-14	21.0
ISG15	205483_s_at	13.1	1.75E-14	22.5
MX1	202086_at	12.2	2.12E-13	20.0
DDX60	218986_s_at	11.4	9.23E-14	20.8
OAS2	204972_at	9.7	1.36E-11	15.9
LY6E	202145_at	7.0	1.80E-11	15.6

^a^Results are from whole blood samples collected from healthy volunteers 6 hours post-dose with 30 μg IM IFN beta-1a. A composite IFN beta induction score was calculated from the geometric mean of the LOD score intensities of the top 20 transcripts.

^b^Values were calculated using F-tests within the software package BRB Array Tools.

^c^LOD score, logarithm (base 10) of odds score.

### PEGylation modifies the kinetics of IFN beta-1a transcriptional response in healthy subjects

**Figure 5:**
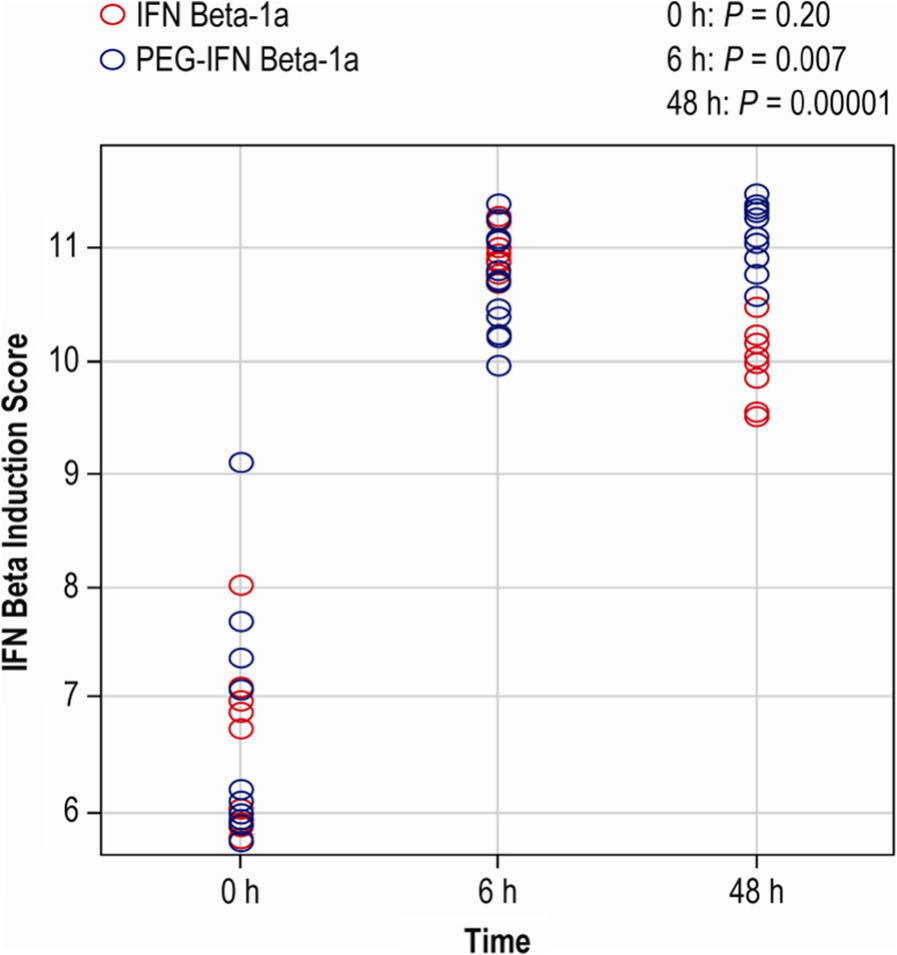
**Differential kinetics of IFN-responsive gene induction by IFN beta-1a and PEG-IFN beta-1a. **Whole blood samples from healthy volunteers were analyzed for expression of established IFN-responsive genes. IFN beta induction scores were calculated as described in the Methods section.

To further demonstrate the practical utility of this new methodology, we next compared the peripheral blood transcriptional response with native IM IFN beta-1a versus PEG-IFN beta-1a in healthy subjects. Healthy volunteers were administered a single dose of IFN beta-1a (30 μg given IM) or PEG-IFN beta-1a (63 μg given IM or subcutaneously [SC]). For comparison of responses to the 2 drugs, composite IFN beta induction scores were calculated from the geometric mean of the normalized intensities of the top 20 induced transcripts at 6 hours post-dose (Table 1). Results showed significant differences between IFN beta-1a and PEG-IFN beta-1a in their induction scores at 6 and 48 hours post-dose (Figure 5), indicating that the up-regulation of IFN-responsive transcripts was longer following dosing with PEG-IFN beta-1a than with IFN beta-1a.

## Discussion

Peripheral blood transcriptional expression profiling is an attractive technology for large pharmacogenomics studies. However, there have been technical limitations to generating robust transcriptional profiles from this important tissue. Although microarray technologies have been standardized and miniaturized to allow much larger numbers of samples to be processed in parallel than was previously possible from tissues and cell lines, there are few robust methods to utilize these highly parallel profiling technologies for the analysis of large numbers of peripheral blood samples. Therefore, development of new methodologies that enable the reproducible generation of expression profiles from thousands of patient blood samples are of paramount importance to translational research.

We report the development and validation of a highly sensitive and reproducible HT whole blood expression profiling methodology, designated BIIB_HT. This methodology can be utilized in conjunction with clinical studies to identify expressed transcripts that may be useful for elucidating drug or disease mechanisms of action, or that can function as prognostic, diagnostic, or toxicity markers. This method was applied to the analysis of whole blood samples collected from healthy clinical trial subjects before and after treatment with a single dose of either IM IFN beta-1a or SC or IM PEG-IFN beta-1a. Study results demonstrate that PEG-IFN beta-1a induces a more sustained and prolonged pharmacodynamic response than unmodified IFN beta-1a. These results provide validation of the utility of this new methodology and support potential therapeutic benefits of PEG-IFN beta-1a.

The BIIB_HT method is unique in that it has been optimized specifically to provide the most robust detection of transcripts from 96 peripheral blood samples in parallel. It can be used to analyze peripheral blood samples from large clinical studies in order to identify expressed transcripts that may be useful for elucidating disease and therapeutic mechanisms of action, as well as for the identification and validation of prognostic, diagnostic, or toxicity markers. BIIB_HT generates sscDNA targets using Ovation® (NuGEN Technologies, San Carlos, CA) amplification technology and has been optimized to provide the maximum sensitivity and specificity when used in combination with the HTA plate array platform from Affymetrix (Santa Clara, CA). sscDNA targets were selected as the amplification moiety for the development of blood profiling methodologies based on internal benchmarking experiments (data not shown) and published reports [21,22]. Because there are currently no reports describing the validation of sscDNA targets for use with the HTA array platform, a systematic optimization was required. Specifically, hybridization and washing conditions and mass/array parameters were optimized using 2 RNA samples from the MicroArray Quality Control (MAQC) project to identify conditions yielding maximum detection and lowest variance sscDNA targets [24]. The current results were referenced against native HTA plate array conditions as well as independent qPCR published results.

TMAC is the native HTA plate array hybridization buffer used with cRNA probes. It has been shown to stabilize adenine-thymine (AT) base pairs (bp) and minimize the effect of base composition on oligonucleotide hybridizations of up to 200 bp. The TMAC hybridization buffer effectively equalizes the melting points of different probes therefore allowing probes with different nucleotide compositions to be hybridized under identical conditions [30,31]. On the other hand, sscDNA hybridizations on glass slide or cartridge arrays typically utilize a 10% DMSO-based buffer. In the presence of DMSO, denatured DNA has been shown to renature with homologous DNA and is not retained by the substrate, thereby reducing the occurrence of background signals [32]. In the current study, the assay results for TMAC-based versus DMSO-based hybridizations were markedly different. Hybridization chemistry (DMSO or TMAC buffer) influenced hybridization quality, as indicated by its ranking as the third most important factor impacting assay results after target type and sample type (Figure 1). In general, use of TMAC-based hybridizations with sscDNA probes produced lower numbers of detectable transcripts and higher background signals across the various masses tested (Figure 2). Given these data, the DMSO-based hybridization buffer was superior to the TMAC-based buffer when sscDNA targets were used in conjunction with the HTA platform.

When assessing possible errors induced as a function of experimental variables, NUSE plots are useful for graphical assessment of array quality. With this method, standard error estimates for each probe set are normalized to a median value of 1 across all arrays. Box plot representation of NUSE values are then drawn for each array and comparative analysis can be conducted for the entire dataset. Arrays or sets of arrays with a larger spread are determined to be of higher variance and are therefore of lower quality. Based on the NUSE plots, use of DMSO buffer with HSH and LSW conditions generated the most reproducible data (Figure 3). Interestingly, mass type and sample type did not strongly influence assay results. These observations may stem from a masking effect due to averaging across sample types. Nevertheless, DMSO was clearly superior to TMAC for use with sscDNA targets on an HT array.

Finally, a PCA in fold change space was used to assess correlations between the variable conditions tested and a “gold standard” qPCR reference dataset. In an effort to normalize all comparisons, qualifiers were selected that were present in the qPCR reference set, changed at least 1.5-fold between HBRR and UHRR datasets, and had a *p-*value of ≤ 0.0001 in any of the conditions tested. This strategy would allow for penalization of any assay conditions generating a false call as well as for benefiting conditions resulting in a correct call. Results showed a clear difference between DMSO and TMAC, with each buffer condition isolated to distinct clusters. Interestingly, the use of DMSO with LSH, HSW, and 2 μg of sscDNA produced results that clustered most closely with the qPCR reference set (Figure 4). Additionally, these optimized conditions markedly outperformed the standard cRNA/HT array hybridization conditions (black data point), suggesting that the methods reported here represent a significant improvement over the current technology. As with the other analyses that were performed, of all the variables tested, the mass of sscDNA for each array had the smallest effect.

Following optimization of the BIIB_HT technical parameters, we sought to apply this new methodology to the analysis of peripheral blood that was collected as part of a clinical trial evaluating administration of IFN beta-1a or PEG-IFN beta-1a to healthy subjects. Human IFN beta-1a is a first-line therapy for patients with relapsing forms of multiple sclerosis (MS). In multiple clinical trials and long-term observational studies, IFN beta-1a has been shown to reduce the development of MS-associated brain magnetic resonance imaging (MRI) lesions, reduce clinical relapse rates, and slow the advancement of physical disability [33-35]. PEG-IFN beta-1a is being developed with the aim of providing a treatment option that is at least as safe and effective as current first-line therapies, but with the added benefits of less frequent dosing and improved convenience. IFN beta-1a was PEGylated by the attachment of a 20 kDa methoxy-PEG-O-2-methylpropionaldehyde to the α-amino group of the N-terminus of IFN beta-1a, a site that is not critical for binding to the type 1 IFN receptor [36,37].

Previous pharmacokinetic studies have shown that after a single parenteral injection, PEG-IFN beta1-a was detectable in peripheral blood after 7 days as compared with 2 days with unmodified IFN beta-1a [29]. This increased drug exposure was accompanied by enhanced and sustained expression of the pharmacodynamic IFN biomarkers 2^′^,5^′^-oligoadenylate synthetase and neopterin. We sought to verify if the transcriptional response would reflect previous observations of a sustained and prolonged pharmacodynamic response to PEG-IFN beta-1a as compared with IFN beta-1a using BIIB_HT assay.

We observed a peak median induction score of 11 at 6 hours post IFN beta1-a dosing. A p-value of 0.007 was calculated by comparison of the IFN induction scores of the 2 groups at 6 hours post dose (Student’s t test). As expected, at 48 hours post-dose the induction score of subjects treated with IFN beta1-1a had declined to 10 while the subjects that received PEG-IFN beta-1a reached a peak Interferon induction score of 11. Again, the p-value that was calculated between the induction scores of the 2 groups was statistically significant (*p* = 0.00001). These transcriptional data reflect the previously reported translational sustained pharmacodynamic response observed with PEG-IFN beta-1a.

## Conclusions

The application of HT microarray technologies to large clinical pharmacogenomics studies represents a unique opportunity to discover prognostic and predictive markers of efficacy and safety on a genome scale. These studies allow a greater understanding of the variable expression of the human transcriptome in response to therapy in a highly controlled setting. A barrier to the execution of these studies is the ability to produce mRNA expression profiles from peripheral blood in a reproducible and robust manner. We believe that the methods presented in this report support the use of HT genome scale expression analysis for biomarker discovery from whole blood samples derived from large clinical trials.

## Methods

### Experimental design

To eliminate confounding factors associated with labeling variances, sscDNA targets were generated in bulk from the highly characterized control RNAs HBRR and UHRR. Following labeling, sscDNAs were hybridized to HT-HGU133A plate arrays using different masses under varying hybridization and washing conditions. These data were used for comparative analyses between the different hybridization conditions as well as against historical HBRR/UHRR cRNA HT array “Genomics” data and 1000 gene “MAQC” qPCR reference data sets to identify optimal hybridization conditions that both maximize detection of rare transcripts and minimize hybridization variance.

HBRR and UHRR sscDNA target masses were titrated from 1.0-2.5 μg/array in DMSO or native TMAC hybridization buffer. Hybridization Stringency was controlled using temperature. High Stringency Hybridizations (HSH) or Low Stringency Hybridizations were conducted at 48-50°C or 41-43°C respectively. Washing stringency was controlled by adjusting the temperature of the stringent wash “B” buffer. High Stringency Washes (HSW) were conducted at 48–50°C and Low Stringency Washes were performed at 41–43°C. Experimental conditions for sscDNA targets were annotated as follows: “Hybridization Cocktail”_“Hybridization Stringency”_“Washing Stringency”. The following conditions were tested for ssDNA targets, DMSO_HSH_LSW, DMSO_HSH_HSW, DMSO_LSH_HSW, TMAC_HSH_LSW, and TMAC_HSH_HSW. All experimental arrays were processed on a GCAS automated workstation using the HYB_01 and WASH_01 protocols (Affymetrix, Santa Clara, CA). Additionally, the results of experimental sscDNA hybridizations were compared with previously published data from both cRNA/HT arrays processed under standard conditions (IVT_Std) and MAQC qPCR reference data sets [24]. All experimental conditions were replicated in triplicate for a total of 120 HT arrays.

### Target preparation and labeling of test RNAs

#### Reference RNAs

Two reference RNAs were used in this study. The Universal Human Reference RNA (catalog number 740000) and Human Brain Reference RNA (catalog number AM6050) samples were purchased from Stratagene/Agilent, Santa Clara, CA and Ambion/Life Technologies, Grand Island, NY respectively.

#### sscDNA target production

sscDNA targets were generated in bulk from 96 replicate 20 ng HBRR or UHRR RNA reactions using the Ovation RNA automated amplification system V2 (catalog number 3100) and the Ovation Whole Blood reagent (catalog number 4200) NuGEN Technologies, San Carlos, CA, according to the manufacturer’s recommendations and then pooled to eliminate any potential confounding factors associated with labeling variance. sscDNA targets were mass titrated and then fragmented using FL Ovation cDNA biotin module (catalog number 4200-A01) NuGEN Technologies, San Carlos, CA according to the manufacture’s recommendation. Finally, fragmented and biotinylated sscDNAs were re-suspended in either TMAC hybridization buffer (100 mM MES. 2.5 M TMAC, 20 mM EDTA, 0.01% Tween-20) or DMSO hybridization buffer (100 mM MES. 1 M [Na+], 20 mM EDTA, 0.01% Tween-20) containing the hybridization controls BioB, BioC, BioD, and cre (P/N 900458, Affymetrix) for HT plates.

#### cRNA target production

The Affymetrix automated Target Preparation protocol (TP_0001) was used to prepare 48 replicates of labeled and unfragmented cRNA in bulk starting from 1 ug of either UHRR or HBRR according to the GeneChip Expression Analysis Technical Manual for Cartridge Arrays using the GeneChip Array Station (catalog number 702064), Affymetrix, Santa Clara, CA. Labeled, unfragmented cRNA yields were calculated for each set of 48 wells and high-quality replicates were then pooled and redistributed to a 96-well plate for manual fragmentation (data not shown). Fragmented cRNA test samples were repooled to achieve uniformity and then split into two aliquots and added to a TMAC hybridization buffer containing the hybridization controls BioB, BioC, BioD, and cre (P/N 900458, Affymetrix) for HT plates.

### Target preparation and labeling of test RNAs

sscDNA targets were generated in bulk from 96 replicate 20 ng HBRR or UHRR RNA reactions using the Ovation RNA automated amplification kit (NuGEN Technologies, San Carlos, CA), and then pooled to eliminate any potential confounding factors associated with labeling variance. sscDNA targets were mass titrated and then fragmented using FL Ovation cDNA biotin module for automation (catalog number 4200-A01). Finally, fragmented and biotinylated sscDNAs were resuspended in either TMAC or DMSO hybridization buffer.

### HT hybridization, washing, scanning, and image processing

sscDNA targets were hybridized to HT plate arrays overnight and then washed and stained as described above. Array images (.dat files) were generated using a GeneChip HT array plate scanner (Affymetrix). Mini “.dat” files were stitched together using the software HT Image Reader, v1.0.27 (Affymetrix). Signal values in “.cel” and “.chp” files and present/absent calls and “.rpt” files containing global array quality metrics were generated for each scanned image using the GCOS Software Statistical Algorithm, v1.0 (Affymetrix). Global quality metrics were imported into Spotfire (Spotfire Inc., Palo Alto, CA) for visualization.

### Clinical study design

Nine subjects (3 females and 6 males) from a phase 1, single-dose, healthy-volunteer, dose and route finding study conducted as part of the clinical development of PEG-IFN beta-1a received a single IM 30-μg injection (6 MIU) of either IFN beta-1a (Avonex®) or PEG-IFN beta-1a 63-μg injection (6 MIU). Peripheral blood samples used for expression profiling were collected prior to injection and at 6 and 48 hours postinjection using the PAXgene Blood RNA System (Qiagen, Hilden, Germany). This study was performed according to the principles outlined in the Declaration of Helsinki and after approval by the Biogen Idec Institutional Review Board.

### Subject information and consent

Prior to any testing under this protocol, including screening tests and assessments, written informed consent with the approved Informed Consent Form (IFC) was obtained from the subject in accordance with local practice and regulations. Written informed consent was obtained from all subjects participating in this clinical study conducted by Biogen Idec.

A copy of the ICF, signed and dated by the subject, was given to the subject. Confirmation of a subject’s informed consent has been documented in the subject’s medical record prior to any testing under this protocol, including screening tests and assessments.

### HT whole blood gene expression profiling

RNA extraction from PAXgene-collected blood samples was conducted using the RNAdvance Blood 96 Well Plate Protocol (Agencourt, Beverly, MA) on an ArrayPlex liquid handling system (Beckman Coulter, Brea, CA). RNA concentration was determined using a Nano-Drop spectrophotometer (Nano-Drop Technologies, Wilmington, NC). sscDNA targets were generated from 20 ng total RNA using the Ovation RNA Automated Amplification Kit (NuGEN Technologies, San Carlos, CA). sscDNA targets (1–2.5 ug) were fragmented using FL Ovation cDNA Biotin Module for Automation (NuGEN Technologies). Fragmented and biotinylated sscDNAs were then resuspended in DMSO hybridization buffer. Blood sscDNA targets were hybridized and washed using the newly optimized experimental conditions. HT plate arrays were hybridized under high stringency conditions (48°C) overnight (16 hours) and washed under low stringency (41°C) conditions. Array images were generated and processed as described above.

### Statistical analyses

All analyses were performed in the R statistical language using BRB ArrayTools v3.6.3 developed by Dr. Richard Simon and Amy Peng Lam. Array quartile normalization and probe set summarizations were performed using the GCRMA procedure as implemented in BRB Array tools. Microsoft excel was employed for calculating standard deviations and coefficients of variation. PCA and hierarchical clustering analysis were performed using Spotfire (http://http:/www.spotfire.com) and R software packages.

## Abbreviations

Bp: Base pair; cDNA: Complementary DNA; cRNA: Complementary RNA; DMSO: Dimethylsulfoxide; HBRR: Human brain reference RNA; HSH: High stringency hybridization; HSW: High stringency wash; HT: High throughput; LSH: Low stringency hybridization; LSW: Low stringency wash; IFN: Interferon; IM: Intramuscular; IVT: *In vitro *transcription; LOD: Logarithm (base 10) of odds; MAQC: Microarray quality control; NUSE: Normalized, unscaled standard error; PCA: Prinicipal component analysis; PEG: Polyethylene glycol; PEG-IFN beta-1a: PEGylated interferon beta-1a; pPCR: Quantitative polymerase chain reaction; SF: Scaling factor; sscDNA: Single-stranded complementary DNA; TMAC: Tetramethylammonium chloride; UHRR: Universal human reference RNA; MIU: Million Units.

## Competing interests

The authors declare that they have no competing interests.

## Authors’ contributions

NEA conceived the study, participated in its design and coordination; carried out RNA sample preparation and qPCR experiments; participated in oligo-array hybridization, data extraction and data interpretation; performed the statistical analyses; and drafted the manuscript. JB, JC, SB, and GB participated in data interpretation and study coordination and contributed to the final draft of the manuscript. All authors read and approved the final version of the manuscript.

## Supplementary Material

Additional file 1 Top induced pathways 6 post Interferon beta administration. Click here for file
